# The Paradoxical Role of Pyroptosis in Gastrointestinal Cancers: From Molecular Mechanisms to Therapeutic Horizons

**DOI:** 10.3390/biomedicines14040911

**Published:** 2026-04-16

**Authors:** John K. Triantafillidis

**Affiliations:** Metropolitan General Hospital, Holargos, 15562 Athens, Greece; info@metropolitan-general.gr or jktrian@gmail.com

**Keywords:** pyroptosis, inflammasome, gasdermin, gastrointestinal cancer, tumor microenvironment, immunotherapy

## Abstract

Pyroptosis, a gasdermin-mediated and highly immunogenic form of regulated cell death, has surfaced as a critical determinant in the progression and therapeutic landscape of gastrointestinal (GI) cancers. Unlike non-inflammatory apoptotic pathways, pyroptosis involves the assembly of inflammasome complexes and the subsequent activation of caspases, leading to the cleavage of gasdermin proteins and the formation of transmembrane pores. It contributes to tumor suppression via immunogenic cell death and activation of antitumor immunity but may also promote tumor progression through chronic inflammation and remodeling of the tumor microenvironment. In this comprehensive review, we delineated the molecular architecture of pyroptotic signaling within the GI tract, highlighting the “double-edged sword” nature of this process. We further evaluated its role in the pathogenesis of GI cancers and in emerging translational strategies, including the pharmacological modulation of gasdermins and microbiome-based interventions, aiming to integrate pyroptosis induction into current immunotherapeutic frameworks.

## 1. Introduction

Gastrointestinal (GI) malignancies represent one of the most significant global health challenges, accounting for approximately one-quarter of all cancer cases and one-third of cancer-related deaths worldwide. In 2022, GI cancers were responsible for an estimated 4.9 million new cases and 3.3 million deaths globally, with projections indicating this burden will nearly double by 2050, reaching 9.06 million incident cases and 6.42 million deaths [1]. The global lifetime risk of developing a GI cancer is 8.20%, translating to one in 12 people developing and one in 16 people dying from these malignancies during their lifetime [2]. The five major GI cancers demonstrate substantial geographic and temporal variations in incidence and mortality. Colorectal cancer (CRC) ranks as the third-most common cancer globally and second in terms of mortality, with over 1.9 million new cases and 904,000 deaths estimated in 2022. The burden is projected to increase to 3.2 million new cases and 1.6 million deaths by 2040, with most cases occurring in countries with a high or very high Human Development Index (HDI). Gastric cancer (GC), the fifth leading cause of cancer mortality, accounts for nearly 1 million new cases and more than 650,000 deaths annually. Esophageal cancer (EC) represents the 11th most prevalent cancer and seventh leading cause of cancer death worldwide, with approximately 510,716 new cases and 445,129 deaths in 2022 [3]. Therefore, malignant diseases of the GI tract represent perhaps the most important part of human malignant neoplasms in terms of constantly changing epidemiological parameters.

The elucidation of the pathophysiological mechanisms of carcinogenesis has been intensively studied in recent years with the aim of improving rational therapeutic treatment. The biological paradigm of cell death has evolved significantly beyond the classical dichotomy of apoptosis and necrosis. Among the recently characterized modalities, pyroptosis stands out due to its unique ability to link intracellular pathogen-sensing with robust extracellular inflammatory signaling. In the context of GI malignancies, the role of pyroptosis is increasingly recognized as both complex and decisive. The GI tract, being a constant interface between the host immune system, dietary antigens, and the dense microbial population, provides a unique niche where pyroptotic events can fundamentally reshape the tumor microenvironment. Understanding how to harness this lytic death process offers the potential to overcome the limitations of current therapies, particularly in “cold” tumors that exhibit resistance to immune checkpoint inhibitors (ICIs).

In this review, we delineated the molecular architecture of pyroptotic signaling within the GI tract, highlighting the “double-edged sword” nature of this process. We further evaluated its role in the pathogenesis of individual GI cancers and in emerging translational strategies. To improve conceptual clarity, we organized the discussion into four major domains: molecular mechanisms, tumor microenvironment interactions, context-specific roles across gastrointestinal malignancies, and therapeutic implications.

## 2. Molecular Mechanisms of Pyroptosis

At the molecular level, pyroptosis is distinguished by its reliance on the gasdermin (GSDM) family of pore-forming proteins, which serve as the final effectors of membrane lysis. The process is predominantly orchestrated through two distinct signaling cascades: the canonical and the non-canonical pathways [4,5,6].

The canonical pathway is initiated by the assembly of multiprotein complexes known as inflammasomes (e.g., NLRP3, AIM2, NLRC4), which respond to a diverse array of pathogen-associated molecular patterns (PAMPs) and danger-associated molecular patterns (DAMPs). Upon activation, these complexes recruit the adaptor protein ASC and pro-caspase-1, leading to the autoproteolytic maturation of caspase-1. This active protease subsequently cleaves the autoinhibitory N-terminal domain of GSDMD and processes pro-IL-1β and pro-IL-18 into their bioactive forms.

**Figure 1:**
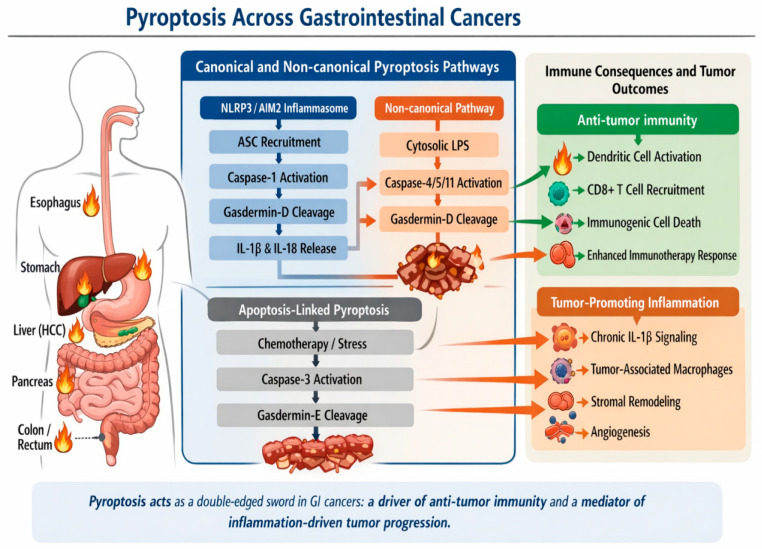
Schematic representation of pyroptosis in GI malignancies: molecular pathways and immune consequences. The figure illustrates both canonical inflammasome-dependent pathways (NLRP3/AIM2–ASC–caspase-1) and non-canonical pathways (caspase-4/5/11 activation by cytosolic lipopolysaccharide), leading to gasdermin-D cleavage and pore formation. These processes result in the release of pro-inflammatory cytokines, particularly interleukin-1β (IL-1β) and interleukin-18 (IL-18). In addition, apoptosis-associated pyroptosis mediated by the caspase-3/gasdermin-E axis is depicted as a mechanism linking therapeutic stress to inflammatory cell death. The downstream consequences of pyroptosis are context-dependent and include both antitumor immune responses—such as dendritic cell activation, CD8+ T-cell recruitment, and enhanced immunogenicity—and tumor-promoting effects, including chronic inflammation, macrophage polarization, stromal remodeling, and angiogenesis. This dual role underscores the complex and dynamic contribution of pyroptosis within the tumor microenvironment of GI cancers.

Non-canonical pathway bypasses traditional inflammasome sensors, involving the direct activation of caspases 4/5 (in humans) or caspase-11 (in mice) by intracellular lipopolysaccharides (LPS) from Gram-negative bacteria. Both pathways converge on the oligomerization of GSDM-N domains within the plasma membrane, disrupting cellular homeostatic gradients and culminating in a lytic, pro-inflammatory demise. Tissue-specific variations modulate the consequences of pyroptosis in different GI cancer types. Figure 1 highlights these two pathways.

### 2.1. Crosstalk Between Apoptosis and Pyroptosis in the Tumor Microenvironment

Emerging evidence highlights a critical crosstalk between apoptosis and pyroptosis, primarily mediated through the caspase-3/GSDME axis. Under specific conditions, caspase-3 activation leads to cleavage of gasdermin E, thereby converting non-inflammatory apoptotic signals into inflammatory pyroptotic cell death. This functional switch has significant implications for tumor immunogenicity and may influence therapeutic responses, particularly in the context of chemotherapy-induced cell death [7]. Within the tumor microenvironment, pyroptosis plays a pivotal role in shaping immune dynamics through the release of cytokines such as interleukin-1β and interleukin-18. These mediators contribute to immune cell recruitment and activation but may also drive chronic inflammation and immune suppression depending on the context. The net effect of pyroptosis on tumor progression is therefore determined by the balance between immune activation and inflammatory dysregulation [8].

Chronic pyroptotic signaling may promote cancer stem-like phenotypes and cellular plasticity, particularly in inflammation-driven tumor environments. This process may contribute to therapy resistance and tumor recurrence, especially in the context of microbiome-associated immune modulation [9].

Pyroptosis contributes to stromal remodeling by modulating extracellular matrix composition and tissue architecture, potentially enhancing drug penetration and therapeutic efficacy. Alterations in stromal permeability may represent an underexplored mechanism linking inflammation to treatment response [10,11].

Nevertheless, pyroptosis should not be interpreted as uniformly tumor-suppressive or tumor-promoting, but rather as a context-dependent process shaped by tumor type, disease stage, and the evolving tumor microenvironment. While acute pyroptotic activation may enhance antitumor immunity, chronic or dysregulated activation may contribute to sustained inflammation and tumor progression. Despite substantial advances in understanding the molecular mechanisms of pyroptosis, its translation into clinical practice remains limited. Most available data are derived from preclinical models, while robust clinical validation is still lacking. Bridging this gap will require well-designed clinical studies and the development of reliable biomarkers to guide therapeutic targeting of pyroptotic pathways.

### 2.2. Inflammasomes as Guardians and Drivers in GI Cancer

In the GI landscape, inflammasomes function as critical sensors at the interface of the host immune system and the gut microbiota. Their role in GI oncogenesis is inherently paradoxical [12]. For instance, the NLRP3 inflammasome has been implicated in both tumor suppression—by fostering immunogenic cell death—and tumor promotion—by sustaining a chronic inflammatory milieu that facilitates epithelial-to-mesenchymal transition (EMT). Specifically, in CRC, the loss of certain inflammasome components has been linked to increased tumor burden, suggesting a protective role during early stages of tumorigenesis. However, as the disease progresses, the persistent release of pyroptosis-derived cytokines may inadvertently remodel the tumor microenvironment (TME) to favor immune evasion and angiogenesis. This context dependency necessitates a nuanced understanding of how inflammasome signaling varies across different GI anatomical sites, from the esophagus to the distal colon.

### 2.3. The Microbiome–Pyroptosis Axis in GI Cancer

The GI tract represents a unique interface where the host immune system continuously interacts with a dense microbial ecosystem. Emerging evidence suggests that certain bacterial species can act as potent modulators of the pyroptotic machinery. For instance, *Fusobacterium nucleatum*, a well-known driver of colorectal oncogenesis, has been shown to manipulate the NLRP3 inflammasome to favor a chronic inflammatory state that promotes tumor cell survival rather than lytic death [13,14]. Conversely, beneficial commensals and their metabolites, such as Short-Chain Fatty Acids (SCFAs), appear to reinforce the intestinal barrier by priming gasdermin-D (GSDMD) expression in healthy epithelial cells, while selectively inducing pyroptosis in malignant cells.

Microbiome profiling could serve as a non-invasive biomarker to predict which patients are more likely to respond to pyroptosis-inducing therapies. In the near future, the integration of designer probiotics engineered to secrete specific inflammasome activators within the gut niche could provide a localized and sustainable method to trigger antitumor pyroptosis while bypassing the systemic side effects of conventional agonists.

### 2.4. The Dichotomous Role of Pyroptosis in Tumor Biology

The role of pyroptosis in cancer biology can be conceptualized as a Yin–Yang dynamic, reflecting its paradoxical capacity to exert both tumor-suppressive and tumor-promoting effects. In early tumorigenesis, pyroptosis may activate innate and adaptive immune responses, thereby contributing to tumor control. In contrast, in advanced disease, chronic pyroptotic signaling may foster a pro-tumorigenic inflammatory milieu, supporting tumor growth, angiogenesis, and metastasis. This functional shift reflects dynamic interactions within the tumor microenvironment, including immune cell recruitment and cytokine signaling. Unlike the immunologically quiescent process of apoptosis, pyroptosis is characterized by cellular swelling, gasdermin-mediated membrane permeabilization, and the catastrophic release of pro-inflammatory cytokines and DAMPs. This inherently inflammatory nature underlies its paradoxical dual role: acting as a potent tumor suppressor by eliminating malignant cells and stimulating antitumor immunity, while simultaneously serving as a tumor promoter by fostering a chronic inflammatory milieu that facilitates genomic instability, angiogenesis, and immune evasion.

### 2.5. Tumor-Suppressive Effects: Lytic Demise and Immune Priming

The primary tumor-suppressive function of pyroptosis lies in its capacity to directly eliminate malignant cells through the formation of gasdermin-dependent transmembrane pores. This highly inflammatory form of cell death (characterized by chromatin fragmentation and cytomembrane lysis) can effectively bypass the traditional resistance mechanisms that tumors develop against apoptosis [15]. A critical axis in this process is the caspase-3/GSDME pathway, where active caspase-3 cleaves GSDME to generate N-terminal fragments that oligomerize and rupture the cell membrane [16,17]. Mechanistically, cancer cell pyroptosis differs significantly from its counterpart in immune cells; it frequently utilizes executioners such as GSDMB, GSDMC, and GSDME, often triggered by apoptotic caspases or granzymes rather than inflammatory caspases [18,19].

Therapeutic modalities including chemotherapy, radiotherapy, and targeted agents can harness these pathways to induce immunogenic cell death. For instance, ionizing radiation has been shown to trigger robust antitumor immunity via the caspase-9/caspase-3/GSDME cascade [20,21]. By modulating the death switch between apoptosis and pyroptosis, clinicians may find new opportunities to overcome treatment-refractory malignancies.

Pyroptosis serves as a bridge between innate and adaptive immunity by releasing potent immunomodulatory signals, including IL-1β, IL-18, HMGB1, and ATP [22,23]. These DAMPs promote the maturation of dendritic cells, enhance tumor antigen presentation, and facilitate the infiltration of cytotoxic T-lymphocytes (CTLs), effectively converting “cold” tumors into “hot” immunogenic microenvironments.

Evidence highlights the role of deubiquitinating enzymes like USP48, which stabilize GSDME to enhance the efficacy of PD-1 inhibitors by revitalizing T-cell and macrophage activity [24]. Furthermore, innovative “cytokine-armed” strategies that combine GSDMD-mediated lysis with the intratumoral expression of IL-12 or IL-18 have demonstrated the potential for systemic, tumor-agnostic immunotherapeutic protection [25].

Conversely, sustained or dysregulated pyroptosis can fuel tumorigenesis by maintaining a pro-inflammatory niche conducive to genomic instability and tumor cell proliferation. The persistent secretion of IL-1β and IL-18 by fibroblasts, macrophages, and tumor cells themselves can remodel the TME to favor initiation and metastasis. This process directly impacts angiogenesis and vascular remodeling, with pan-cancer analyses indicating that high-pyroptosis subgroups are often enriched in oncogenic pathways such as EMT, IL6-JAK-STAT3, and PI3K-AKT-mTOR [26].

Strategic immune evasion is another hallmark of chronic pyroptosis. Within a hypoxic TME, HIF-1α signaling can modulate inflammasome activity to promote the recruitment of immunosuppressive populations, such as myeloid-derived suppressor cells (MDSCs) and regulatory T-cells (Tregs) [10]. Notably, aberrant GSDME expression in certain contexts, like HCC, has been shown to facilitate immune suppression through pyroptosis-independent mechanisms [27]. Furthermore, some persister cancer cells can develop resistance by utilizing methionine flux and taurine production to maintain membrane integrity and silence immune-response genes through DNA hypermethylation [28].

## 3. Context-Specific Roles Across GI Malignancies

The types of GI cancer most associated with the involvement of pyroptosis are gastric, colorectal, and esophageal cancer. Pyroptosis has also been implicated in pancreatic ductal adenocarcinoma, hepatocellular carcinoma, and gallbladder cancer, but the strongest evidence and most detailed mechanistic studies focus on gastric and colorectal cancers, where pyroptosis-related genes and inflammasome activation play a significant role in tumor progression, immune microenvironment modulation, and prognosis.

### 3.1. Esophageal Cancer

The involvement of pyroptotic pathways in esophageal malignancies, particularly Esophageal Squamous Cell Carcinoma (ESCC) and adenocarcinoma (EAC), represents a complex interplay between chronic epithelial irritation and inflammatory signaling. In ESCC, pyroptosis often functions as a potent tumor-suppressive mechanism. Specifically, the activation of the NLRP3 inflammasome and subsequent GSDMD-mediated lysis has been shown to inhibit cellular proliferation and trigger systemic antitumor signaling [29]. However, the esophageal microenvironment is uniquely influenced by exogenous factors such as gastroesophageal reflux and microbial dysbiosis, which can chronicize inflammasome activation. This chronic state may inadvertently promote a pro-tumorigenic niche through the persistent secretion of IL-1β, facilitating esophageal mucosal remodeling and malignant transformation. Evidence highlights the potential of pharmacological agents to reactivate “silenced” pyroptotic genes in EC cells, offering a synergistic approach when combined with traditional chemotherapeutic regimens [30].

### 3.2. Gastric Cancer (GC)

In GC, pyroptosis serves as a pivotal bridge between *Helicobacter pylori* (*Hp*) infection and gastric oncogenesis. The *Hp*-induced activation of the NLRP3/caspase-1 axis is a hallmark of the initial inflammatory response, yet its progression toward malignancy is often characterized by the strategic downregulation of pyroptotic effectors [31]. For instance, GSDME silencing via promoter hypermethylation is frequently observed in GC, allowing tumor cells to evade immunogenic death and maintain proliferative capacity [32]. Restoring GSDME expression through epigenetic modulators or specific targeted therapies has demonstrated remarkable efficacy in sensitizing GC cells to apoptosis-to-pyroptosis switching. Furthermore, the prognostic significance of a pyroptosis-related gene signature in GC is becoming increasingly evident, as it correlates with the infiltration of M1-type macrophages and CD8+ T-cells, thereby defining a more favorable immune landscape and predicting better responses to ICIs.

### 3.3. Colorectal Cancer (CRC)

In the landscape of colorectal carcinogenesis, pyroptosis functions as a critical homeostatic rheostat. The transition from adenoma to carcinoma is frequently characterized by the strategic evasion of GSDMD-mediated cell death, often orchestrated by the dysregulation of the NLRP3 inflammasome. In the colonic epithelium, pyroptosis not only serves as a barrier against malignant transformation by eliminating damaged cells but also acts as a potent orchestrator of the immune landscape [33]. Furthermore, the role of long non-coding RNAs (lncRNAs) and microRNAs in silencing pyroptotic effectors has emerged as a key mechanism for tumor survival in CRC [34]. Therefore, pyroptosis seems to play a dual role in immune modulation and immunogenic cell death.

Regarding the clinical implications, the expression levels of GSDME and NLRP3 in biopsy specimens could serve as valuable biomarkers for stratifying patients’ risk of recurrence [35]. Moreover, inducing an apoptosis-to-pyroptosis switch could enhance the efficacy of 5-Fluorouracil-based regimens. Research is currently focusing on leveraging the gut–vascular barrier to deliver pyroptosis-inducing nanoparticles directly to CRC lesions, aiming to achieve a localized immunogenic effect without exacerbating systemic colitis.

### 3.4. Hepatocellular Carcinoma (HCC)

HCC typically arises within a background of chronic inflammation, where pyroptosis plays a paradoxical role. While the canonical NLRP3/caspase-1 axis is often downregulated in advanced HCC to avoid immune recognition, its over-activation during the early stages of hepatitis or cirrhosis may contribute to the fibrotic remodeling of the liver niche [36]. Interestingly, the downregulation of GSDMD has been correlated with poor differentiation and a high proliferative index in HCC cells, suggesting that bypassing pyroptosis is a hallmark of hepatic malignancy.

Pharmacological agents that reactivate GSDME-mediated death could sensitize HCC to multi-kinase inhibitors like Sorafenib, potentially overcoming primary resistance. The development of targeted therapies that specifically trigger pyroptosis in hepatocytes through the non-canonical pathway (caspase-4/5) represents a promising frontier for precision hepatology, particularly in “cold” HCC tumors.

### 3.5. Cholangiocarcinoma (CCA)

CCA remains one of the most treatment-refractory GI malignancies, characterized by an exceptionally dense and immunosuppressive stroma. Pyroptosis in CCA is often inhibited through epigenetic modifications, which prevents the release of immunogenic DAMPs that could otherwise activate tumor-infiltrating lymphocytes. The interplay between bile acid-induced stress and inflammasome signaling is a unique feature of CCA, where certain bile acids can either trigger or suppress pyroptotic events depending on the biliary microenvironment [37,38].

Targeting the pyroptotic threshold in biliary epithelial cells may offer a way to disrupt the dense stroma and allow better penetration of chemotherapeutic agents. Investigating the crosstalk between pyroptosis and the biliary microbiome could lead to the discovery of specific bacterial metabolites that serve as natural inducers of gasdermin-mediated death in CCA.

### 3.6. Pancreatic Ductal Adenocarcinoma (PDAC)

PDAC is notorious for its fibrotic desmoplasia and immune evasion. In this context, pyroptosis offers a revolutionary strategy to “re-heat” the tumor microenvironment. By activating the caspase-3/GSDME axis, it is possible to transform the typically silent apoptotic response into a lytic, pro-inflammatory event. This shift not only eradicates the primary tumor cell but also releases a cascade of cytokines that can mobilize cytotoxic T-cells into the previously impenetrable pancreatic stroma [39].

Regarding the clinical implications, it seems that combining gemcitabine or nab-paclitaxel with small-molecule pyroptosis activators could fundamentally change the standard of care for PDAC by converting non-responders into responders [40]. Future clinical trials are expected to evaluate bi-specific molecules that simultaneously inhibit oncogenic pathways and activate gasdermins, aiming for a synergistic eradication of pancreatic cancer cells and their surrounding supportive niche.

In summary, current evidence on pyroptosis in GI cancers is derived predominantly from mechanistic and preclinical studies, while robust clinical validation remains limited. This gap underscores the need for cautious interpretation when extrapolating experimental findings into therapeutic strategies. Importantly, biomarker-driven studies suggest potential clinical relevance; however, heterogeneity across tumor types and study designs limits direct translational applicability.

Nevertheless, it is clear that pyroptosis plays a complex and context-dependent role in GI malignancies. Across esophageal, gastric, colorectal, hepatocellular, pancreatic, and biliary tract cancers, inflammasome activation serves as a central mechanism linking chronic inflammation to carcinogenesis. The inflammasome pathway, through caspase-1 activation and subsequent cleavage of gasdermins, leads to pore formation in cell membranes and release of pro-inflammatory cytokines IL-1β and IL-18. This process exhibits a dual nature: while acute pyroptosis can suppress tumor growth by eliminating malignant cells and activating antitumor immunity, chronic inflammasome activation promotes tumor progression through sustained inflammation, immune evasion, and remodeling of the tumor microenvironment.

Gasdermin expression patterns vary significantly across GI cancers and carry important prognostic implications. GSDME emerges as a particularly significant family member, demonstrating tumor-suppressive functions in several cancer types while also mediating chemotherapy-induced pyroptosis. GSDMD exhibits context-dependent roles, functioning as a tumor suppressor in CRC while promoting progression in HCC. GSDMC and GSDMB are frequently associated with poor prognosis, promoting tumor cell proliferation, invasion, and immune evasion. The silencing of certain gasdermins through epigenetic mechanisms, particularly DNA methylation, represents a common feature across multiple GI malignancies, suggesting potential therapeutic opportunities through demethylating agents.

The clinical relevance of pyroptosis in GI cancers extends to prognosis prediction and therapeutic response. Pyroptosis-related gene signatures successfully stratify patients into distinct risk groups with significant survival differences across all GI cancer types examined. Low pyroptosis risk scores are generally associated with enhanced antitumor immune infiltration, higher microsatellite instability, and improved response to ICIs. Furthermore, specific etiological factors unique to each cancer type, including *Hp* infection in GC, chronic viral hepatitis and cirrhosis in HCC, bile acid dysregulation in cholangiocarcinoma, and gut microbiota alterations in CRC, converge on inflammasome activation as a common pathogenic mechanism. Therapeutic strategies targeting the pyroptosis pathway, including NLRP3 inhibitors, gasdermin modulators, and combination approaches with immunotherapy, represent promising avenues for improving treatment outcomes in GI malignancies.

## 4. Therapeutic Implications

Pharmacological modulation of pyroptosis is rapidly becoming a cornerstone of innovative GI cancer therapeutics. Current strategies focus on two primary objectives: the direct induction of gasdermin-mediated death in tumor cells and the inhibition of pro-tumorigenic chronic inflammation. Small molecules that trigger the apoptosis-to-pyroptosis switch are particularly promising, as they can overcome the immune-evasive nature of traditional apoptotic death. Furthermore, the combination of pyroptosis inducers with ICIs has demonstrated synergistic effects, where the release of DAMPs and cytokines from pyroptotic cells re-arms the T-cell population within the TME.

In regard to the clinical implications, therapeutic success in this field will likely require a personalized approach, where the baseline expression of gasdermins in a patient’s tumor biopsy guides the choice of drug combinations. Next-generation delivery systems, such as antibody–drug conjugates or stimuli-responsive nano-carriers, are being developed to deliver pyroptotic payloads specifically to the malignant site, thereby maximizing the immunogenic effect while shielding healthy GI tissues from inflammatory damage.

### 4.1. Pharmacological Inhibition of Inflammasome Signaling

While inducing pyroptosis is a primary anti-neoplastic strategy, the selective inhibition of inflammasome signaling is equally vital in mitigating tumor-promoting chronic inflammation [20,41]. Examples include (i) NLRP3 inhibitors, representing small molecules targeting the NLRP3 ATPase activity (e.g., MCC950), which have demonstrated efficacy in dampening the cytokine storm within the TME, potentially preventing the epithelial-to-mesenchymal transition in GI cancers, and (ii) caspase-1 and IL-1β blockade via monoclonal antibodies targeting IL-1β (e.g., Canakinumab) or selective caspase-1 inhibitors aimed to decouple cell death from harmful systemic inflammation. Regarding the clinical implications, it seems that in patients with high baseline levels of systemic inflammation, inflammasome inhibitors may serve as an adjuvant therapy to stabilize the TME before initiating aggressive cytotoxic regimens.

### 4.2. Synergizing Pyroptosis with Immunotherapy

The integration of pyroptosis induction with ICIs represents a paradigm shift in cancer immunotherapy. Pyroptosis acts as an immunogenic catalyst; by converting the cold non-responsive TME into a hot environment rich in DAMPs and pro-inflammatory cytokines, it facilitates the recruitment of CD8+ T-cells and dendritic cells [21]. Regarding future strategies, engineering bi-specific agents that can simultaneously block PD-1/PD-L1 pathways and trigger gasdermin-mediated pores could overcome the primary resistance observed in a significant portion of GI cancer patients.

### 4.3. Microbiome-Targeted Interventions

The gut microbiota serves as a distal regulator of the pyroptotic threshold. Strategic interventions using specific probiotics or microbial metabolites (such as butyrate) can prime the inflammasome response in tumor cells while maintaining mucosal integrity. The development of precision symbiotics designed to activate the non-canonical caspase-4/11 pathway offers a novel, microbiome-based approach to trigger selective pyroptosis in CRC and GC. Sustained pyroptotic signaling has been increasingly associated with the acquisition of cancer stem-like properties and enhanced cellular plasticity. In inflammation-driven tumor environments, particularly those influenced by the gut microbiome, pyroptosis-related pathways may promote therapy resistance and tumor recurrence. This emerging link further reinforces the complex and multifaceted role of pyroptosis in cancer biology.

### 4.4. The Utility of Biomarkers

The clinical utility of pyroptosis is underscored by its potential as a reservoir for novel biomarkers. Regarding diagnostic potential, elevated circulating levels of IL-1β, IL-18, and HMGB1 are being evaluated as minimally invasive liquid biopsy signatures for the early detection of GI malignancies. In regard to prognostic significance, the robust expression of GSDMD or GSDME in surgical specimens is increasingly correlated with improved overall survival and reduced lymph node metastasis, as it reflects a higher capacity for immunogenic cell death [22]. Identifying predictive markers is essential for personalizing pyroptosis-based therapies. The epigenetic status of gasdermin promoters (e.g., methylation levels) can predict whether a tumor will undergo silent apoptosis or immunogenic pyroptosis in response to chemotherapy. This could have significant clinical implications because implementing pyroptosis-related gene signatures in clinical practice will allow clinicians to stratify patients into responders and non-responders for both conventional chemotherapy and next-generation immunotherapies [23,42].

## 5. Conclusions and Future Perspectives

Pyroptosis represents a context-dependent mechanism in gastrointestinal cancers, with its biological impact varying according to tumor type, stage, and microenvironmental signals. While early-stage activation may promote antitumor immunity through immunogenic cell death, chronic activation may instead sustain tumor-promoting inflammation and immune evasion.

Crosstalk between apoptosis and pyroptosis has emerged as a critical regulatory axis within the tumor microenvironment. Caspase-3-mediated cleavage of gasdermin E enables a functional switch from apoptosis to pyroptosis, thereby enhancing immunogenicity and influencing tumor repopulation dynamics following therapy.

Pyroptosis also contributes to stromal remodeling, influencing extracellular matrix composition and tumor stiffness. This may enhance drug penetration and represents a potential therapeutic strategy across multiple gastrointestinal malignancies.

Importantly, sustained pyroptotic signaling has been linked to cancer stem-like phenotypes and cellular plasticity, particularly in inflammation-driven and microbiome-influenced tumor environments. This duality further reinforces the concept of pyroptosis as a double-edged sword in cancer biology.

Therefore, pyroptosis, far from being a mere alternative to apoptosis, represents a sophisticated regulatory hub that bridges innate immunity with adaptive antitumor responses. A deeper understanding of its molecular regulation and interactions within the tumor microenvironment will be essential for harnessing its therapeutic potential. The transition from the basic molecular mapping of gasdermin-mediated lysis to its clinical application in GI cancers is currently at a critical juncture. While the double-edged sword nature of pyroptosis, balancing between antitumor immunity and pro-tumorigenic chronic inflammation, remains a challenge, it also provides a unique therapeutic window.

Despite substantial progress, several key questions remain unanswered. The context-dependent role of pyroptosis, the lack of standardized biomarkers, and the complexity of inflammasome signaling networks pose significant challenges. Future research should focus on combination therapies, personalized inflammasome targeting, precision oncology and pyroptosis, defining molecular signatures of pyroptosis in different GI tumors, developing selective modulators of gasdermin activation, and conducting well-designed clinical trials evaluating inflammasome-targeted therapies.

We posit that the future of GI cancer treatment will not rely on a “one-size-fits-all” induction of cell death, but rather on the spatial and temporal precision of pyroptosis activation. The most promising frontier lies in the development of smart delivery systems that can trigger the apoptosis-to-pyroptosis switch exclusively within the tumor microenvironment, thereby avoiding systemic cytokine release syndrome. Furthermore, integrating pyroptosis-related gene signatures into routine pathological assessment could revolutionize patient stratification, identifying those who would derive the greatest benefit from combined pyroptotic and immune checkpoint blockade.

## Data Availability

No new data were created or analyzed in this study.

## Abbreviations

The following abbreviations are used in this manuscript:

GI	Gastrointestinal
IL	Interleukin
CRC	Colorectal cancer
HDI	Human Development Index
GC	Gastric cancer
EC	Esophageal cancer
EAC	Esophageal adenocarcinoma
CSCC	Esophageal Squamous Cell Carcinoma
ICIs	Immune checkpoint inhibitors
GSDM	Gasdermin
PSMPs	Pathogen-associated molecular patterns
DAMPs	Danger-associated molecular patterns
LPS	Lipopolysaccharides
EMT	Epithelial-to-mesenchymal transition
TME	Tumor microenvironment
SCFAs	Short-Chain Fatty Acids
CTLs	Cytotoxic T-lymphocytes
MDSCs	Myeloid-derived suppressor cells
Tegs	Regulatory T-cells
Hp	Helicobacter pylori
HCC	Hepatocellular carcinoma
CGA	Cholangiocarcinoma
PDAC	Pancreatic Duct Adenocarcinoma
